# Psychological distress and associated factors among cancer patients in public hospitals, Addis Ababa, Ethiopia: a cross-sectional study

**DOI:** 10.1186/s40359-023-01079-5

**Published:** 2023-02-10

**Authors:** Frehiwot Negussie, Berhanu Wordofa Giru, Nete Tewfik Yusuf, Debela Gela

**Affiliations:** 1 Cancer Center of Tikur Anbessa Specialized Hospital, Addis Ababa, Ethiopia; 2 School of Nursing and Midwifery, College of Health Sciences, Addis Ababa University, 4412 Addis Ababa, Ethiopia

**Keywords:** Psychological distress, Distress, Cancer, Adult, Addis Ababa, Ethiopia

## Abstract

**Background:**

Cancer has great implications for psychological, social, economic, and emotional dimensions. Psychological distress is overwhelming among cancer patients following a confirmed diagnosis. However, little is known about the prevalence of psychological distress and associated factors among cancer patients in Africa Sub-Saharan. Thus, this study aimed to assess the prevalence of psychological distress and associated factors among cancer patients in public hospitals in Addis Ababa, Ethiopia.

**Methods:**

An institution-based cross-sectional study was conducted among cancer patients from September 15, 2019, to June 30, 2020. A total of 386 cancer patients selected through a simple random sampling technique participated in the study. Data were collected by an interview-administered questionnaire to evaluate psychological distress with a distress thermometer and social support with the Oslo 3-items Social Support Scale. The collected data were entered into Epi-data version 4.2 and exported into SPSS 25 for analysis, and then binary and multivariate logistic regressions were done to identify the association between dependent and independent variables.

**Results:**

A total of 386 study participants were included in the study with a response rate of 91.4%. The prevalence of psychological distress among cancer patients in public hospitals in Addis Ababa, Ethiopia was 64.5%. Age > 45 years [AOR = 0.41; 95% CI (0.22–0.77)], marital status of being divorced [AOR = 3.3; 95%CI (1.23–8.71)] and married [AOR = 3.2; 95%CI (1.03–10.40)], rural residence [AOR = 1.5; 95%CI (1.15–5.18)], cancer stage II [AOR = 3.9; 95%CI (1.90–15.50)], stage III [AOR = 3.5;95%CI (1.45–8.44)] and stage IV [AOR = 3.4; 95%CI (1.90–10.11)], co-morbidity [AOR = 0.07; 95%CI: (0.03–0.17)], and moderate social support [AOR = 0.36; 95%CI (0.14–0.60)] and strong social support [AOR = 0.06; 95%CI (0.03–0.12)] were found to be significantly associated with psychological distress.

**Conclusion:**

The prevalence of psychological distress among cancer patients in public hospitals in Addis Ababa, Ethiopia was high, and age, marital status, place of residence, cancer stage, co-morbidity, and social support were associated with psychological distress. Therefore, interventions focusing on these findings require special emphasis during designing interventions aimed at decreasing psychological distress.

## Background

Cancer is a disease condition caused by uncontrolled cell growth and division; as a result, it damages the immune system, a network of organs, tissues, and specialized cells that protect the body from infections as well as other psychosocial function impairments [1]. It remains one of the principal causes of death and concern that do not respect geographical, economic, ethnic, or social barriers, especially in Africa, which remains poorly prepared and gives less priority to cancer epidemics [2]. In Sub-Saharan Africa, the incidence of cancer is still rising alongside infectious disease conditions, which are related to low attention to non-communicable diseases, including cancer, low economic status, and scarce diagnostic treatment modalities [3–6].

Distress ranges from normal fears, worry, and sadness to disabling problems such as clinical depression, generalized anxiety, panic, isolation, or a spiritual or existential crisis [7]. As a result of distress symptoms and psychiatric problems, maladaptive coping and abnormal illness behavior occur [3]. Psychological distress and psychosocial problems that occur due to cancer diagnosis or cancer progression can be expressed as unpleasant emotional or psychological experiences, including depression, anxiety, adjustment disorders, delirium, and other/mood disorders [8–14].

Globally, cancer is a major public health problem that causes significant psychological problems for patients and families due to its increasing incidence [3, 15]. It causes devastating emotional and financial distress and overall distress [16]. Moreover, it causes a substantial problem for clients so that families or caregivers involved in end-of-life care suffer from psychological morbidity [17]. Psychological morbidity is higher among patients than among family caregivers or the general population [18, 19]. The incidence of diagnosis of psychological problems rises significantly, which accounts for 97.5% of cancer cases who seek help for mental health [20, 21].

Psychological factors that affect the uptake of screening and investigations, decision-making, and adherence to treatment or those factors that negatively impact trust and relationships with a healthcare team can overwhelm a person’s available resources and significantly affect the person's quality of life (QoL), lowering future life satisfaction and the lives of their families and carers [22–24]. In Ethiopia, it is estimated that the incidence of cancer cases was 21,563 and 42,722 among males and females, respectively, predominantly breast cancer in females and colorectal cancer and non-Hodgkin's lymphoma in males. The incidence has increased due to the increased prevalence and aging population alongside an increasing adoption of cancer-causing behaviors such as smoking, alcohol, and dietary habits [25–27]. Despite the ministry of health proposing continuous measurement of the progress and impact of cancer control activities in about 23% of health facilities offering services for cancer cases, the psychological problems cancer patients face are not fully addressed in Africa Sub-Saharan [28]. Hence, the purpose of this study is to assess the prevalence and associated factors of psychological distress among cancer patients in public hospitals in Addis Ababa, Ethiopia, in 2020.

## Methods and materials

### Study area and duration

This study was conducted in the oncology unit of public hospitals in Addis Ababa, Ethiopia, which was reserved for inpatient and outpatient oncology services. Of those hospitals, Tikur Anbessa Specialized Hospital (TASH) is the only oncology center in Addis Ababa, Ethiopia that gives chemotherapy, surgery, radiation, and comprehensive services to clients. There were five clinical oncologists, seven radiotherapists, nine oncology nurses, and 18 nurses working at the oncology units. TASH aspires to become a center of excellence in the diagnosis, treatment, and care of patients with cancer. With the support of Ethiopia's governmental institutions, non-governmental organizations, and international partners, including the International Network for Cancer Treatment and Research (INCTR), the hospital is hoping to develop a comprehensive cancer care program, including a cancer registry, early detection, prevention, standard treatment, and palliative care [29]. The study was conducted from September 15, 2019, to June 30, 2020.

### Study design and population

An institution-based cross-sectional study was employed. All randomly selected adult (18 years and older) cancer patients from the oncology unit of public hospitals during the study period were included, except cancer patients who were critically ill (those cancer patients who were unable to communicate during the data collection time due to being critically ill).

### Sample size determination and sampling procedure

The sample size of this study was determined using a single population proportion formula by considering the following statistical assumptions: prevalence of psychological distress as 50% since there was no study done in Ethiopia regarding psychological distress among cancer patients, 95% confidence interval, and 5% margin of error. The final sample size, including the non-response rate, yielded 422 cancer patients. To select the eligible study subjects, the number of patients followed per week was determined. Hence, 465 patients were attending the oncology department per week. On average, a total of 1,860 cancer patients attend the oncology unit within one month. A total of 422 cancer patients were selected from 1860 reference populations using a simple random sampling technique.

### Study variables

The dependent variable was psychological distress and the independent variables included sociodemographic factors (age, gender, residence, marital status), clinical and treatment factors (type of cancer, stage, duration of illness, comorbidity, treatment), and socioeconomic factors (monthly income, social support, educational status, occupation).

### Data collection tools and procedures

The data were collected using Amharic version structured interview questionnaires. First, the questionnaire was prepared in the English language, then translated into the Amharic language. Six nurses holding a Bachelor of Science degree were involved in data collection under the supervision of two Master of Science degree students. The training was provided by the principal investigator for the data collectors and supervisors for one day before the data collection on the objective of the study, the measuring tool, and participant recruitment strategies. The questionnaire consisted of socio-demographic factors, clinical-treatment factors, and socioeconomic factors. Patients' charts were assessed if patients did not know some variables like the stage, site of cancer, and receipt of treatment. Stages of cancer range from stage 0–IV based on the American Joint Committee on Cancer (AJCC) [30].

A distress thermometer is an effective distress screening instrument for cancer patients. It is a less time-consuming, accepted, and validated tool with a specificity rate of 0.68 to 0.78 and a sensitivity rate that ranges from 0.65 to 0.77. It is a visual analog scale that runs from 0 (no distress) to 10 (extreme distress), with a score of > 5 considered as psychological distress and a score of less than 5 considered as no psychological distress. The checklist has five categories, such as practical, physical, family, emotional, and spiritual categories [31].

Oslo 3-items The Social Support Scale (OSS-3) is an important instrument to screen for social and social-related problems. OSS-3 covers different fields of social support and is put together into a composite index of social support by summing up the standardized Z-scores for each item. The sum index is made by summarizing the raw scores, the sum ranging from 3 to 14. Patients with poor support had a 3–8 score, moderate support had a 9–11 score, and strong support had a 12–14 score [32].

### Data processing and analysis

The collected data was cleaned, coded, and then entered using Epi-data version 4.2 and then exported into SPSS version 25 for analysis. Basic descriptive analyses were done in terms of central tendency and dispersion value for continuous data and frequency distribution for categorical data based on the natural distribution. Bivariate and multiple logistic regressions were done using a 0.05 level of significance to determine the association of each independent variable with psychological distress. The *p*-value < 0.05 in the multivariable analysis, was considered statistically significant. The results were expressed as an adjusted odds ratio (AOR) with a 95% confidence interval, and *p*-values were used to measure the association and to identify statistically significant factors of psychological distress.

## Results

### Socio-demographic and socio-economic characteristics of the study participants

A total of 386 participants participated in the study, with a response rate of 91.4%. Of the study participants, 72.8% were females. All the participants were between the ages 18–91 with a mean age of 45 ± 13.56 standard deviation years. The majority of the participants were ≤ 45 years (*n* = 215, 55.7%). One-third of participants (33.9%) resided in rural areas. Nearly half of the study participants, 47.9% had educational status in secondary and higher institution programs. Of the study participants, 37.1% of the study participants were employed. The average monthly income of study participants was 2960.8 ± 4459.8 SD ETB and 45.3% of the participants had poor social support (Table 1).

**Table 1:** Socio-demographic characteristics of a study participant in public hospitals, Addis Ababa, Ethiopia 2020 (*n* = 386)

Variable	Category	Frequency (*n*)	Percent (%)
Gender	Male	105	27.2
	Female	281	72.8
Age (in years) Mean (SD) = 45 ± 13.56	≤ 45	215	55.7
	> 45	171	44.3
Marital status	Single	69	17.8
	Married	223	57.8
	Divorced	44	11.4
	Widowed	50	13.0
Residence	Urban	255	66.1
	Rural	131	33.9
Monthly income (ETB)	< 500	104	26.9
	501–1000	64	16.6
	> 1001	218	56.5
Educational status	Can`t read and write	66	17.1
	Primary	135	35.0
	Secondary	100	25.9
	College and university	85	22.0
Occupation	Unemployed	19	4.9
	Employed	143	37.1
	Housewife	100	25.9
	Farming	48	12.5
	Daily laborer	43	11.1
	Other*	33	8.5
Social support	Poor support	175	45.3
	Moderate support	132	34.2
	Strong support	79	20.5

*ETB* Ethiopian Birr

*Merchant, Student, Retire

### Clinical characteristics of the study participants

Among the study participants, 162 (42%) had breast cancer. Approximately two-thirds (69.9%) of study participants had late-stage cancer at the time of data collection. Of those, 36.0% had a clinical stage IV. The average length of diagnosis was 17.3 + 19.2 SD months. Nearly half of the participants, 184 (47.7%), had received chemotherapy (Table 2).

**Table 2:** Clinical characteristics of study participants in public hospitals, Addis Ababa Ethiopia, 2020(*n* = 386)

Variable	Category	Frequency(*n*)	Percent (%)
Cancer type	Lung cancer	28	7.3
	Breast cancer	162	42.0
	Colorectal cancer	71	18.4
	Cervical cancer	42	10.8
	Sarcoma	28	7.3
	Esophageal cancer	23	6.0
	Gastric cancer	11	2.8
	Others*	21	5.4
Cancer stage	Stage I	45	11.7
	Stage II	71	18.4
	Stage III	131	33.9
	Stage IV	139	36.0
Length of diagnosis	< 12 months	193	50.0
	> 12 months	193	50.0
Co-morbidity	Yes	135	35.0
	No	251	65.0
Treatment received	Radiation	7	1.8
	Surgery	9	2.3
	Chemotherapy	184	47.7
	Surgery plus chemotherapy	118	30.6
	Radiation plus surgery plus chemotherapy	34	8.8
	Surgery plus radiation	34	8.8

*Lymphoma Ca, Prostate Ca, Testicular Ca, Leukemia Ca, Endometrial Ca, Vulvar Ca, Skin Ca, Laryngeal Ca, Nasopharyngeal Ca, Orbital Ca, Oral Ca, Pancreatic Ca, Squamous Cell Carcinoma

### Prevalence of psychological distress

The prevalence of psychological distress among cancer patients was 64.5%**.** The mean score of psychological distress was 5.44 ± 2.0SD.

### Factors associated with psychological distress

In bivariable analysis, age, marital status, place of residence, monthly income, educational status, cancer stage, co-morbidity, and social support were associated with psychological distress among study participants. In multivariable analysis, age, marital status, place of residence, cancer stage, co-morbidity, and social support were associated with psychological distress.

Study participants over the age of 45 years [AOR = 0.41; 95% CI (0.22–0.77)] were 59% less likely to develop psychological distress than those with an age ≤ 45 years. Those study participants who divorced [AOR = 3.3; 95%CI (1.23–8.71)] and married [AOR = 3.2; 95%CI (1.03–10.40)] were 3.3 and 3.2 times more likely to develop psychological distress compared to a single one, respectively. Study participants living in rural areas were 1.5 times more likely to have psychological distress compared with those living in urban areas [AOR = 1.5; 95%CI (1.15–5.18)]. Study participants who had cancer stage II [AOR = 3.9; 95%CI (1.90–15.50)], stage III [AOR = 3.5;95%CI (1.45–8.44)], and stage IV [AOR = 3.4; 95%CI (1.90–10.11)] were 3.9, 3.5 and 3.4 times more likely to developed psychological distress than those diagnosed at stage I, respectively. Study participants who did not have co-morbid conditions were 93.0% less likely to have psychological distress than those with co-morbid conditions [AOR = 0.07; 95%CI: (0.03–0.17)]. Study participants who had moderate social support [AOR = 0.36; 95%CI (0.14–0.60)] and strong social support [AOR = 0.06; 95%CI (0.03–0.12)] were 64.0% and 94.0% less likely to have psychological distress than those who had poor social support, respectively (Table 3).

**Table 3:** Factors associated with psychological distress among cancer patients in public hospitals, Addis Ababa, Ethiopia, 2020 (*n* = 386)

Variable	Category	Psychological distress		COR (95%CI)	AOR (95%CI)
		Yes	No		
Age	≤ 45	128	87	1	1
	> 45	121	50	0.61 (0.39–0.93)	0.41 (0.22–0.77)*
Marital status	Single	37	32	1	1
	Married	133	90	5.3 (2.10–13.40)	3.2 (1.03–10.40)*
	Divorced	36	8	4.1 (1.71–9.61)	3.3 (1.23–8.71)*
	Widowed	43	7	1.3 (0.45–4.10)	1.2 (0.44–5.32)
Residence	Urban	153	102	1	1
	Rural	96	35	1.8 (1.15–2.90)	1.5 (1.15–50.18)*
Monthly Income (ETB)	≤ 500	68	36	1.0 (0.67–1.41)	0.8 (0.37–1.94)
	501–1000	36	28	1.5 (0.87–2.7)	2.5 (0.91–7.09)
	≥ 1001	145	73	1	1
Educational status	Can`t read and write	43	23	0.57 (0.29- 1.12)	0.82 (0.31–2.15)
	Primary	87	48	0.59 (0.34- 1.02)	0.7 (0.30–1.55)
	Secondary	75	25	0.35 0.19- 0.66)	0.15 (0.16–1.77)
	College and university	44	41	1	1
Cancer stage	Stage I	21	24	1	1
	Stage II	40	31	5.4 (2.60–11.31)	3.9 (1.90–15.50)*
	Stage III	73	58	3.7 (1.91–7.06)	3.5 (1.45–8.44)*
	Stage IV	115	24	3.8 (2.21–6.61)	3.4 (1.90–10.11)*
Co-morbidity	Yes	117	18	1	1
	No	132	119	0.17 (3.09–0.29)	0.07 (0.03–0.17)*
Social support	Poor	148	27	1	
	Moderate	73	59	0.4 (0.25–0.78)	0.36 (0.14–0.60)*
	Strong	28	51	0.12 (0.05–0.18)	0.06 (0.03–0.12)*

*p-value < 0.05

*AOR* Adjusted Odds Ratio; *COR* Crude Odds Ratio; *CI* Confidence Interval

## Discussion

This study explored the prevalence and factors associated with psychological distress among cancer patients in public hospitals in Addis Ababa, Ethiopia, and found that 64.5% of cancer patients had psychological distress. The current finding was higher than the studies conducted in South Korea (28.5%) [33], China (39.5%) [34], Taiwan (22.1%) [35], Southern India (38.5%) [36], and Australia (7.5%) [37], but it was lower than the studies conducted in Iran (67.7%) [38] and Germany (89.3%, 84.3%) [39, 40]. This discrepancy might be due to differences in the scale used (other researchers used the Hospital Anxiety and Depression Scale (HADS), and in this study, the distress thermometer was used to measure psychological distress), the length of time that the study participants were enrolled, the cutoff point for the distress thermometer's visual analog score, and the presence of different associated factors that precipitate psychological distress. Another possible reason might be due to the difference in sample sizes, income (e.g., minor resources to address all the needs of the patients, minor infrastructures, and public transport to reach the hospital), various situations (social and cultural differences), different cancer types, and the variability in the mental-health system [41, 42].

This study showed that, compared with younger study participants (≤ 45 years), older study participants (> 45 years) were less likely to develop psychological distress. This finding goes together with other studies done in Saudi Arabia [43], Taiwan [35], and Germany [44]. This might be related to the fact that as age increases, experience, copying mechanisms, and ways of handling stress increase [44]. Thus, younger cancer patients may be in particular need of psychological support, promotion of their well-being, and interventions that will increase their resilience and help lessen the psychological distress during cancer treatment.

Our study found that study participants who were married and divorced were more likely to experience psychological distress than those who were single. This finding goes together with the studies done in Saudi Arabia [43], China [34], Southeast Asia [45], and India [36]. The reason that being married makes one more likely to develop psychological distress might be due to holding more responsibility for their home, family, and child care and an imbalance of demand for and supply of resources to their family while being paired. Divorce increases the likelihood of developing psychological distress, which may be due to its effects on stress stimulators, social support, and health-related behavior [38, 46–50]. Therefore, married and divorced participants need psychological support and promotion of their well-being to lessen their psychological distress during cancer treatment.

This study revealed that residing in rural areas was found to be a significant factor in the development of psychological distress. This finding was similar to a previous study conducted in Iran [38] and Athens [51]. This might be related to the fact that participants residing in rural areas have poor access to health care services, low perception of lifestyle modification, relaxation, and recreation for the management of distress, and low seeking behavior to get psychological and mental health counselors or consultations [41, 52–54]. Thus, giving special attention to the participants who reside in rural areas is essential. This is done by bringing community-based health education to their dwellings.

In this study, the cancer stage was found to be significantly associated with psychological distress. Study participants who had cancer at stages II-IV were more likely to develop psychological distress than those diagnosed at stage I. This result was similar to other previous studies done in Southeast Asia [45], Athens [51], and India [55]. This might be related to the fact that cancer patients who had advanced stages of the disease (stages II-IV) had severe signs and symptoms of the disease, and the patients are aware that a disease can metastasize and requires adhering to the different types of treatments (radiotherapy, chemotherapy, and/or surgery) [41, 44, 54, 56]. Therefore, emphasizing cancer patients with late stages of the disease is required when designing interventions aimed at decreasing psychological distress. In addition to these, the government should give special emphasis to developing a comprehensive cancer control program that includes prevention, early detection, standard treatment, and palliative care.

The findings of this study showed that the presence of co-morbid conditions was found to be significantly associated with psychological distress, which was similar to other studies done in Germany, South Korea, and Iran [38, 57, 58]. This could be due to symptom burden, and multiple medication use related to the presence of co-morbid conditions, which might cause distress regardless of social support, income, socioeconomic, clinical-pathological, and socio-demographic factors [44, 59]. Thus, participants with co-morbid conditions require psychotherapy to decrease their psychological distress.

In this study, social support was also found to be significantly associated with psychological distress. Study participants who had poor social support were more likely to have psychological distress than those who had moderate or strong social support. This concept goes together with the studies done in Germany [58], Turkey [60], South India [36], and South Africa [61]. This might be related to the fact that poor social support with an increasing disease stage could be related to decreased relationships within social circles and decreased coping mechanisms [57]. Therefore, participants with poor social support need support services or psychosocial care to lessen their psychological distress during cancer treatment.

### Strengths and limitations of the study

This study has a couple of strengths. One, it is the first study on psychological distress among cancer patients in Addis Ababa, Ethiopia. Secondly, the study used validated standardized questionnaires and a reasonable sample size. Our study also has some limitations. Firstly, the study is cross-sectional, which means it cannot determine the causation or temporality of the association between associated factors among cancer patients. Prospective and experimental studies are warranted. Secondly, the study used an interviewer-administered structured questionnaire for data collection. Using this method to identify psychological distress and associated factors among cancer patients might involve some risk of concealing information, though qualitative interviews can let participants liberally highlight their concerns about psychological distress.

## Conclusion

This study revealed that the prevalence of psychological distress among cancer patients in Addis Ababa public hospitals was high. According to our results, cancer patients presented a high level of psychological distress during their therapeutic process, especially for specific subgroups of patients (e.g., young age (≤ 45 years), divorced or married participants, patients residing in rural areas, having an advanced cancer stage (stages II-IV)). Therefore, promotion of cancer patient's well-being and prevention of their psychological distress are needed to address the encountered mental health difficulties in cancer patients during oncological treatment. In addition to these, the government should give special attention to developing a comprehensive cancer control program that includes prevention, early detection, standard treatment, and palliative care.

## Data Availability

The datasets used and/or analyzed during the current study are available from the corresponding author upon reasonable request.

## Abbreviations

ACS: American cancer society
AJCC: American joint committee of cancer
Ca: Cancer
CI: Confidence interval
ETB: Ethiopian Birr
HADS: The Hospital Anxiety and depression scale
IRB: Institutional review board
INCTR: International network for cancer treatment and research
AOR: Adjusted odd ratio
NCI: National Cancer Institute
OSS-3: Oslo 3 social support scale
SPSS: Statistical package for social science
SSA: Sub Saharan Africa
TASH: Tikur Anbessa Specialized Hospital
US: United States
WHO: World Health Organization
